# Transjugular Portosystemic Stent Shunt: Impact of Right Atrial Pressure on Portal Venous Hemodynamics Within the First Week

**DOI:** 10.1007/s00270-021-03003-z

**Published:** 2021-12-01

**Authors:** Michael Bernhard Pitton, Arndt Weinmann, Roman Kloeckner, Jens Mittler, Christian Ruckes, Christoph Düber, Gerd Otto

**Affiliations:** 1 Department of Diagnostic and Interventional Radiology, University Medical Center, Johannes Gutenberg University Mainz, Langenbeckstr.1, 55131 Mainz, Germany; 2 Department of Internal Medicine, University Medical Center, Langenbeckstr.1, 55131 Mainz, Germany; 3 Department of General and Visceral Surgery and Transplantation Surgery, University Medical Center, Langenbeckstr.1, 55131 Mainz, Germany; 4 Present Address: Interdisciplinary Center for Clinical Trials (IZKS), University Medical Center, 55131 Mainz, Germany; 5 Emeritus of the Division of Transplantation Surgery, University Medical Center, Langenbeckstr.1, 55131 Mainz, Germany

**Keywords:** Liver cirrhosis, Portal hypertension, Transjugular portosystemic stent shunt (TIPS), Portosystemic pressure gradient (PSG)

## Abstract

**Purpose:**

Porto-systemic pressure gradient is used to prognosticate rebleeding and resolution of ascites after TIPS. This study investigates the reliability of portal pressure characteristics as quantified immediately after TIPS placement and at short-term control.

**Patients and Methods:**

Portal venous pressure (PVP) and right atrial pressure (RAP) were prospectively obtained before and after TIPS as well as ≥ 48 h after TIPS procedure. Porto-systemic pressure gradients (PSG) and pressure changes were calculated. A multivariate regression analysis was performed to predict portal hemodynamics at short-term control.

**Results:**

The study included 124 consecutive patients. Indications for TIPS were refractory ascites, variceal bleeding or combinations of both. Pre- and post-interventional PSG yielded 16.4 ± 5.3 mmHg and 5.9 ± 2.7 mmHg, respectively. At that time, 105/124 patients (84.7%) met the target (PSG ≤ 8 mmHg). After 4 days (median), PSG was 8.5 ± 3.5 mmHg and only 66 patients (53%) met that target. In patients exceeding the target PSG at follow-up, PVP was significantly higher and RAP was lower resulting in the increased PSG. The highly variable changes of RAP were the main contributor to different pressure gradients. In the multivariate regression analysis, PVP and RAP immediately after TIPS were predictors for PSG at short-term control with moderately predictive capacity (AUC = 0.75).

**Conclusion:**

Besides the reduction of portal vein pressure, the highly variable right atrial pressure was the main contributor to different pressure gradients. Thus, immediate post-TIPS measurements do not reliably predict portal hemodynamics during follow-up. These findings need to be further investigated with respect to the corresponding clinical course of the patients.

**Supplementary Information:**

The online version contains supplementary material available at 10.1007/s00270-021-03003-z.

## Introduction

Portal hypertension is the crucial pathophysiological finding in end-stage liver cirrhosis and is responsible for clinical complications such as refractory ascites and variceal bleeding [1, 2]. Portal pressure gradients exceeding 10 mmHg are defined as clinically significant portal hypertension [3]. Transjugular Portosystemic Stent Shunt (TIPS) has shown to reduce portal venous pressure (PVP) and porto-systemic pressure gradients (PSG) [4–8] and has been used for risk stratification regarding variceal bleeding and refractory ascites [3, 9, 10]. The diameter of the stent shunts is adjusted to pre-defined pressure levels [11, 12] which have to be balanced against hepatic encephalopathy [13].

In clinical practice, we noticed considerable deviations from portal pressure gradients taken immediately after TIPS compared to control measurements after a few days. Patients undergoing TIPS procedures obviously feature varying hemodynamic conditions depending not only on the degree of cirrhosis but also on factors such as pre-existing spontaneous porto-venous collaterals, extent of fluid overload, use of diuretics, cardiac pre-conditions and others. The immediate pressure relief after TIPS creation therefore only reflects the instantaneous hemodynamic changes after opening the shunt but does not reflect portal pressure load after equilibration during further follow-up [14]. This study was performed to quantify those time-dependent effects on portal hemodynamics and to predict future deviations from the intended PSG at follow-up.

## Methods

### Study Population

**Fig. 1:**
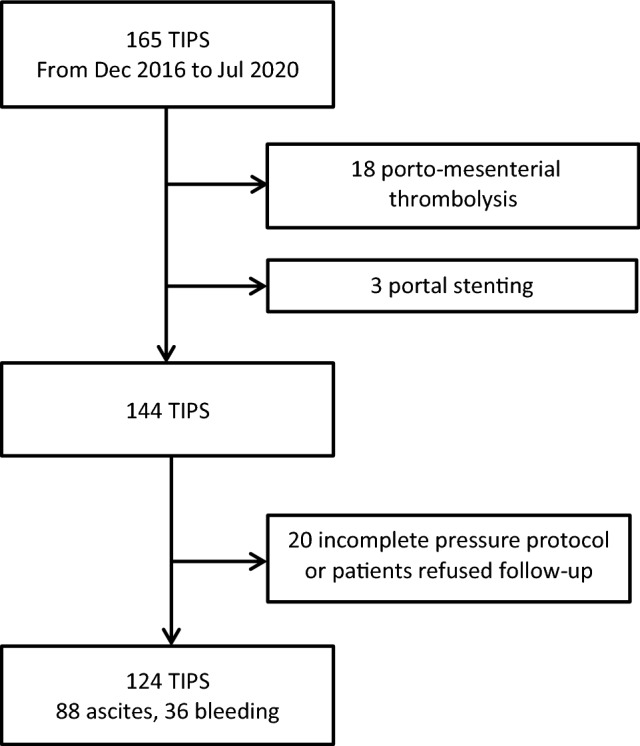
Flowchart of patient selection

Between November 2017 and July 2020, a total of 165 patients were treated with TIPS at our tertiary referral center. Patients with TIPS for porto-mesenterial thrombosis were excluded due to a lack of reliable initial pressure values. Patients who refused short-term follow-up and patients with incomplete pressure protocol were also excluded. Finally, a total of 124 patients with complete pressure measurement, including pre-TIPS and immediate post-TIPS data, and short-term follow-up entered this study (Fig. 1). The study was approved by the local ethics committee (Rhineland Palatinate Ethics Committee, Germany, 15582).

### TIPS Technique

The basic technique for TIPS has been introduced in the 1990s [10] and has been adapted during the years. All TIPS procedures were performed under general anesthesia by two experienced interventional radiologists (MBP, RK). After transjugular approach, the 10F sheath was advanced into the inferior vena cava. The right hepatic vein was catheterized and the sheath was advanced into the right hepatic vein. A flexible trocar stylet was advanced until the tip entered the right portal vein (RUPS-100, Cook). TIPS was created via standard access (*n* = 119), left hepatic vein to left portal vein (*n* = 2), middle hepatic vein to right (*n* = 1) and left portal vein (*n* = 1), and direct transcaval to right hepatic vein (*n* = 1).

Pressure measurements were performed using a 5F-pigtail catheter positioned in the main portal vein, whereas the tip of the 10F-sheath remained in the right atrium. Mean portal vein pressure (PVP) and mean right atrial pressure (RAP) were registered, and the porto-systemic pressure gradient (PSG) was calculated by the difference of both. Measurements were obtained before (PVPpre, RAPpre) and immediately after TIPS (PVPpost, RAPpost), as well as at short-term follow-up (PVPcontrol, PARcontrol). At these points of time, pressure changes were calculated by subtraction of the respective values, immediately after TIPS (Δ post–pre) and at follow-up (Δ control-post). All registrations were performed in expiratory arrest, both during general anesthesia pre- and immediately post-TIPS. Measurements at follow-up were obtained without any sedation under controlled expiratory arrest avoiding any Valsalva effects. Measurements were simultaneously obtained with continuous double line registration of PVP and RAP pressure curves using two parallel electromechanical transducers (LogiCal®, Smiths Medical) and a dedicated workstation (Axiom Sensis XP, Siemens). The system was calibrated at the level of the right atrium against the surrounding atmosphere and set to 0 mmHg. Pressure registrations were obtained for at least 10 s to allow for equilibration of the pressure curves of PVP and RAP and to avoid any Valsalva effects.

PTFE-covered stentgrafts were used (Viatorr® and ViatorrCx®, Gore) for TIPS. In variceal bleeding, the respective varices and large volume porto-systemic shunts were embolized. The target for post-TIPS PSG was defined as PSG ≤ 8 mmHg. In patients with a PSG > 8 mmHg after TIPS, the stentgrafts were adapted by further balloon dilation. In cases in whom this cut-off could not be achieved, no further action was taken at that point. Patients were supervised on the intermediate or intensive care unit and set on heparin according to the underlying co-morbidities and previous coagulation status. Short-term follow-up was scheduled not before 48 h after TIPS creation. Patients who fulfilled the cut-off of ≤ 8 mmHg at short-term follow-up (PSGcontrol) were allocated to group I, and patients who failed this cut-off were allocated to group II.

### Statistical Methods

Statistical calculations were performed using SPSS, version 26 and SAS, Version 9.4. Analysis included descriptive demographic patient data and hemodynamic data before and immediately after TIPS as well as respective hemodynamic data at follow-up. Multivariate regression analysis was performed in order to identify predictors for PSG at short-term follow-up. *P* values ≤ 0.05 were considered significant. Based on significant predictors, the sum of the products of the regression coefficients multiplied by the respective individual values was calculated. Receiver operating characteristics (ROC) and areas under the ROC curves (AUROC) were calculated to evaluate the accuracy of risk prediction. Optimal cut-off values for predictors were determined by maximizing the score test statistic.

## Results

A total of 124 patients were included in this prospective register study, 81 male, 43 female, age 58.2 ± 13.1 years (Table1). Patients were treated because of refractory ascites (*n* = 78), variceal bleeding (*n* = 34) or a combination of both (*n* = 12). Before TIPS creation, PVP and RAP were 24.7 ± 5.2 and 8.3 ± 4.6 mmHg, respectively, resulting in a PSG of 16.4 ± 5.3 mmHg. After TIPS creation, PVP dropped to 17.6 ± 4.6 mmHg, RAP rose to 11.7 ± 4.4 mmHg yielding a PSG of 5.9 ± 2.7 mmHg. At short-term follow-up, PVP was further reduced to 15.0 ± 5.1 mmHg and RAP equilibrated to 6.4 ± 4.6 mmHg resulting in a respective increase of PSG of 8.5 ± 3.5 mmHg (Table 2). At this point, patients were allocated in two groups, those who met the target criterion of PSGcontrol ≤ 8 mmHg (group I) and those who did not (group II). The median interval for short-term follow-up was 4 days (detailed data of follow-up intervals are available in Suppl.Tab.1 and 2).

**Table 1:** Demographics

Demographics	*n*	%
Patients (n)	124	
Age (years; mean ± SD)	58.3 ± 13.4	
Male / Female (n)	81/43	65.3/34.7
*Clinical stage*		
Child–Pugh (A/B/C)	12/78/43	9.7/62.9/27.4
Child–Pugh points Median (Q1/Q3)	9 (8/10)	
MELD Median(Q1/Q3)	13 (10/15)	
NaMELD Median(Q1/Q3)	16 (12/20)	
*Etiology of underlying liver disease*		
Alcohol	75	60.5
Viral hepatitis	7	5.6
Budd-Chiari syndrome	7	5.6
PBC/PSC	2	1.6
NASH	7	5.6
Cryptogenic/others	26	21.0
*Clinical indication for TIPS*		
Refractory ascites / hydrothorax	78	62.9
Refractory ascites + history of bleeding	10	8.1
Variceal bleeding	34	27.4
Variceal bleeding + ascites	2	1.6
*Concomitant findings of cirrhosis*		
Esophageal varices (grade I/II/III/IV)	94 (34/38/18/4)	75.1 (27.4/30.6/14.5/3.2)
Previous treatment of varices	67	54.0
Rectal hemorrhoidal varices	4	4.8
HE total (grade I-II/grade III-IV)	24 (15/9)	19.4 (12.1/7.3)
Hepatorenal syndrome	38	60.6
Spontaneous bacterial peritonitis	22	17.7
Hypersplenic syndrome	11	8.9
Hepatic hydrothorax	13	10.5
Hypertensive gastropathy	49	39.5
*Additional co-morbidities*		
Cardiac diseases	26	21.0
Coronary heart disease	12	9.7
Valvular heart disease	1	0.8
Myocardial insufficiency	9	7.3
Combination of these / others	4	3.2
Arterial Hypertension	42	33.9
Chronic pancreatitis	3	2.4
Polyneuropathy	3	2.4
Diabetes mellitus	33	26.6
Pumonary diseases	15	12.1
Congenital coagulopathy	5	4.0
Hypo-/Hyperthyroidism	15	12.1
Other diseases	24	19.4

Laboratory test	Median (Q1/Q3)	Range
INR	1.3 (1.2/1.4)	1.0–2.2
Creatinine (mg/dl)	1.03 (0.77/1.36)	0.45–4.92
Bilirubin (mg/dl)	1.30 (0.80/2.24)	0.30–12.80
Albumin (g/l)	27.0 (23.0/31.0)	1.0–40.0
Thrombocytes (n/µl)	118 (73/201)	25–679

**Table 2:** Portal hemodynamics before and after TIPS creation. Total: all 124 patients. Group I and II: post hoc allocation of patients to both groups depending on whether patients fulfilled or failed the PSG target at follow-up (PSGcontrol)

Portal Hemodynamics	PVPpre	RAPpre	PSGpre	PVPpost	RAPpost	PSGpost	PVPcontrol	RAPcontrol	PSGcontrol
Total	24.7 ± 5.2 (13 to 48)	8.3 ± 4.6 (− 3 to 27)	16.4 ± 5.3 (3 to 38)	17.6 ± 4.6 (6 to 31)	11.7 ± 4.4 (2 to 26)	5.9 ± 2.7 (0 to 14)	15.0 ± 5.1 (3 to 29)	6.4 ± 4.6 (− 2 to 18)	8.5 ± 3.5 (1 to 20)
Group I	23.3 ± 4.3 (13 to 33)	7.7 ± 3.9 (− 3 to 20)	15.6 ± 4.8 (3 to 31)	16.4 ± 4.6 (6 to 29)	11.5 ± 4.4 (2 to 26)	4.9 ± 2.4 (1 to 14)	13.2 ± 5.0 (3 to 26)	7.2 ± 5.0 (0 to 19)	6.0 ± 1.8 (1 to 8)
Group II	26.3 ± 5.7 (16 to 48)	9.1 ± 5.2 (0 to 27)	17.2 ± 5.7 (6 to 38)	18.9 ± 4.2 (12 to 31)	12.0 ± 4.4 (4 to 24)	6.9 ± 2.5 (0 to 13)	17,0 ± 4.3 (10 to 29)	5.6 ± 3.8 (− 2 to 16)	11.5 ± 2.5 (9 to 20)
*p*	< 0.01	0.092	0.096	0.02	0.507	< 0.001	< 0.001	0.051	< 0.001

*Group I*, cut-off PSG < 8 mmHg at short-term TIPS control fulfilled; *Group II*, cut-off PSG failed at short-term TIPS control; *P*, significance level comparing group I and II, T-Test; *PVP*, Portal vein pressure; *RAP*, Right atrial pressure; *PSG*, Porto-systemic pressure gradient; *PVP/RAP/PSG pre*, pressure levels before *TIPS*; *PVP/RAP/PSGpost*, pressure levels immediately after TIPS; *PVP/RAP/PSGcontrol*, pressure levels at short-term follow-up

Considering all 124 patients, 105 of 124 patients (84.7%) primarily fulfilled the cut-off immediately after TIPS. At follow-up, only 66 patients (53%) still met that cut-off (group I), whereas 58 patients (46.8%) did not (group II, Table 4). The post hoc analysis of pressure data showed that compared to group I, group II presented with significantly higher PVP before and after TIPS, as well as at TIPS control (Table 2). A separate subgroup analysis of those 105 patients who primarily met the cut-off is provided as supplement data. However, there were no discernible relevant differences between the baseline criteria of those patients who subsequently met or did not meet the cut-off PSG at short-term follow-up (Suppl.Tab.3).

**Fig. 2:**
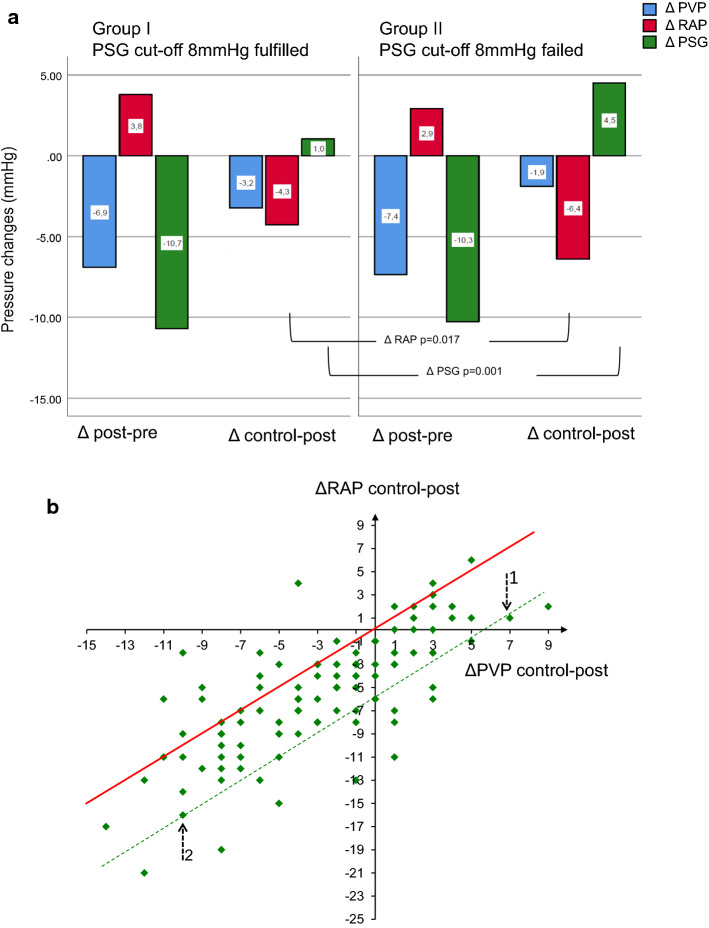
Pressure changes following TIPS. **a** ΔPVPpost-pre (blue), ΔRAPpost-pre (red), ΔPSGpost-pre (green): Mean pressure changes immediately after TIPS compared to pre-interventional measurement. ΔPVPcontrol-post, ΔRAPcontrol-post, ΔPSGcontrol-post: Mean pressure changes at short-term follow-up compared to immediate post-TIPS measurement. Group I: Cut-off PSG ≤ 8 mmHg at short-term follow-up fulfilled. Group II: Cut-off PSG failed at short-term follow-up. **b** Dotplot of the distribution of individual pressure changes at short-term follow-up (ΔPVPcontrol-post and ΔRAPcontrol-post); 4 dots lacking because of identical characteristics at post-TIPS and follow-up measurements. Cases localized above the oblique red line had a further decrease in PSG at follow-up compared to immediate post-TIPS measurement with diverse ΔPVP and ΔRAP. Cases on the red line had identical PSG after TIPS and at follow-up but different ΔPVP and ΔRAP. Cases below the red line had a further increase of PSG at follow-up compared to immediate post-TIPS. Two examples (green dotted line): Example 1: ΔPVP +7 mmHg, ΔRAP +1 mmHg = ΔPSG +6 mmHg compared to PSG after TIPS. Example 2: ΔPVP -10 mmHg, ΔRAP -16 mmHg = ΔPSG +6 mmHg compared to PSG after TIPS despite an absolute reduction of PVP

Pressure changes during follow-up were significantly different between group I and II. In particular, group II presented with reduced ΔPVPcontrol-post and a greater ΔRAPcontrol-post, resulting in a significantly greater ΔPSGcontrol-post at follow-up (Table 3, Fig. 2a). The volatility and the greater amounts of changes of the right atrial pressure was the main contributor to the different PSG levels in patients who failed the cut-off at follow-up. At follow-up, PSGcontrol increased in100 patients compared to after TIPS. In 67 of these patients, the increase in PSG was associated with an absolute decrease in PVP and was thus caused by an even greater drop in RAP (Fig. 2b).

**Table 3:** Individual changes of portal hemodynamics (ΔPVP, ΔRAP, ΔPSG). Δ*post-pre*, pressure difference between the post-TIPS and pre-TIPS values. Δ*control-post,* pressure difference between TIPS control and immediate post-TIPS values

	ΔPVPpost-pre	ΔRAPpost-pre	ΔPSGpost-pre	ΔPVPcontrol-post	ΔRAPcontrol-post	ΔPSGcontrol-post
Group I	− 6.9 ± 3.9 (− 18 to 2)	3.8 ± 3.5 (− 3 to 21)	− 10.7 ± 4.7 (− 28 to 0)	− 3.2 ± 4.7 (− 14 to 5)	− 4.3 ± 4.7 (− 17 to 6)	1.1 ± 2.6 (− 8 to 5)
Group II	− 7.4 ± 5.1 (− 34 to 2)	2.9 ± 3.8 (− 8 to 11)	− 10.3 ± 5.1 (− 28 to 1)	− 1.9 ± 4.2 (− 12 to 9)	− 6.4 ± 5.0 (− 21 to 3)	4.5 ± 3.2 (− 3 to 12)
*p*	0.583	0.198	0.643	0.1	0.017	0.001

*Group I*, cut-off PSG < 8 mmHg at short-term TIPS control fulfilled; *Group II*, cut-off PSG failed at short-term TIPS control, *P*, significance level comparing group I and II, T-Test

If using a different cut-off for PSGcontrol of ≤ 10 mmHg or even ≤ 12 mmHg, the respective post-TIPS cut-offs were met in 118 and 122 cases, respectively. However, at short-term TIPS control, even these cut-offs were fulfilled in only 94 and 104 cases, respectively (Table 4).

**Table 4:** **a** Number of patients who met the cut-off of PSG ≤ 8 mmHg (Group I) or failed the cut-off (Group II) at short-term follow-up (PSGcontrol). **b** Number of patients who met or failed the cut-off of PSG ≤ 10 mmHg **c** Number of patients who met or failed the cut-off of PSG ≤ 12 mmHg

	PSGcontrol ≤ 8 mmHg Group I	PSGcontrol > 8 mmHg Group II	
(a)			
PSGpost ≤ 8 mmHg	60	45	105 (84.7%)
PSGpost > 8 mmHg	6	13	19 (15.3%)
	66 (53%)	58 (46.8%)	124 (100%)

	PSGcontrol ≤ 10 mmHg	PSGcontrol > 10 mmHg	
(b)			
PSGpost ≤ 10 mmHg	91	27	118 (95.2%)
PSGpost > 10 mmHg	3	3	6 (4.8%)
	94 (75.8%)	30 (24.2%)	124 (10%)

	PSGcontrol ≤ 12 mmHg	PSGcontrol > 12 mmHg	
(c)			
PSGpost ≤ 12 mmHg	102	20	122 (98.4%)
PSGpost > 12 mmHg	2	0	2 (1.6%)
	104 (83.9%)	20 (16.1%)	124 (100%)

*PSGpost*, PSG immediately after TIPS; *PSGcontrol*, PSG at short-term follow-up

**Fig. 3:**
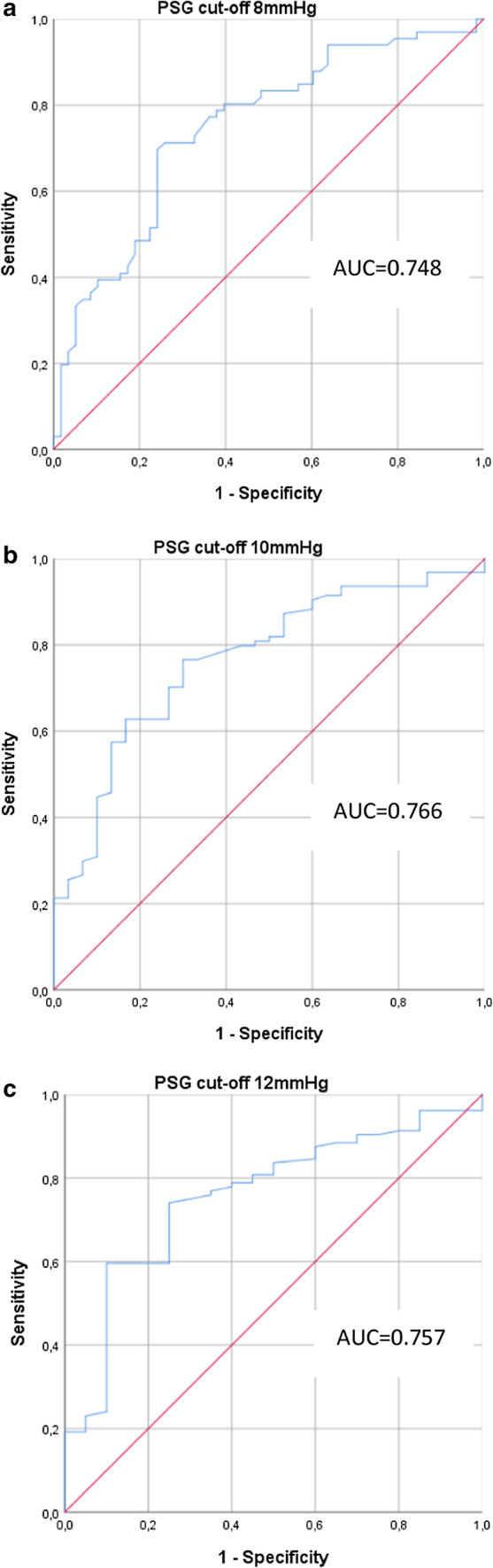
ROC analysis of the risk factors and calculation of AUC for the target PSG at short-term follow-up. Results for PSGcontrol ≤ 8 mmHg (Fig. 3a), PSGcontrol ≤ 10 mmHg (Fig. 3b), and PSGcontrol ≤ 12 mmHg (Fig. 3c)

In order to test whether unexpected pressure levels at short-term follow-up would be predictable, all pre- and post-operative pressures (PVP, RAP, PSG) and respective pressure changes were entered into a binary multiple regression analysis. PVPpost and RAPpost after TIPS placement proved to be the best predictors for meeting the targeted PSG at follow-up (PSGcontrol). The ROC analysis yielded only a moderate predictive capacity (AUC = 0.748) and sensitivity and specificity of 69.7% and 75.8%, respectively. Likewise, the AUC values for the follow-up PSG cut-off ≤ 10 mmHg or ≤ 12 mmHg were again only moderately predictive (Table 5, Fig. 3).

**Table 5:** ROC analysis of the risk factors and calculation of AUC for the target PSG at short-term follow-up (PSGcontrol)

	AUC	Intercept	PVPpost coefficient	RAPpost coefficient	Optimal cut-off	Sensitivity	Specificity
PSGcontrol ≤ 8 mmHg	0.748	3.3947	− 0.3769	0.2879	− 3.1511	69.7%	75.8%
PSGcontrol ≤ 10 mmHg	0.766	4.8949	− 0.3549	0.2297	− 3.9240	76.6%	70.0%
PSGcontrol ≤ 12 mmHg	0.757	4.9863	− 0.3044	0.1907	− 3.1135	59.6%	90.0%

*PVPpost*, portal venous pressure, immediately after TIPS; *RAPpost*, right atrial pressure, immediately after TIPS

## Discussion

This study investigates the changes of portal hemodynamics during short-term follow-up after TIPS creation. Our data show that the PSG obtained immediately after shunt creation does not represent the hemodynamics during further follow-up. Instead, a dynamic equilibration of the pressure values is to be expected resulting in considerable proportion of patients out of the desired pressure range. In particular, the pressure changes of the right atrium were significantly different after TIPS and give an insight into the pathophysiology of different PGS resultants during follow-up. This is of particular interest because the right atrium, the zero point of cardiopulmonary circulation, acts retrograde on portal pressure after TIPS creation. This includes that different portal pressures might result in the same pressure gradient. Vice versa, a particular portal pressure might result in diverse portal pressure gradients.

Silva-Junior et al. have already reported on timing effects of portal pressure gradients after TIPS placement [14]. The authors reported either reduced or even increased immediate pressure gradients depending on whether TIPS was performed under general anesthesia or in deep sedation. Their data were retrospectively collected over 8 years at seven institutions and covered different conditions concerning emergencies versus elective procedures. The major drawback of that publication is, however, the lack of precise data explaining those PSG changes over time. It is of crucial interest to show if changes in PSG are caused by altering PVP or by RAP. In contrast, our data were prospectively collected and are based on simultaneous registration of PVP, RAP, and PSG as well as individual pressure changes over time. Thereby, the specific cut-off level for the target PSG itself is of minor impact on our general statement that the immediate PSG should not be used for risk prediction or further decision-making regardless of the targeted PSG (8, 10, or 12 mmHg). Clinical guidelines with respective recommendations based on a certain PSG immediately measured after TIPS should be questioned.

The pathophysiology of venous return is very complex, particularly in cirrhotic patients. As defined by Guyton, venous return (VR) is the result of the pressure gradient between the mean systemic filling pressure (MSFP) and the right atrial pressure (RAP) divided by the resistance to venous return (RVR) (VR = MSFP-RAP/RVR). According to this equation, venous return increases with high MSFP and low right atrial pressure [15–17]. In a clinical setting, only RAP and not factors such as volume load, elastic distension of the veins, sympathetic tone, cardiac function, drugs etc. can reliably be obtained during a TIPS procedure. The venous return and the potential backpressure from the right atrium into the liver veins cover a variety of pathophysiological effects that need for equilibration during follow-up and potentially impact the further clinical course. After TIPS placement, the situation is characterized by the reduced porto-venous pressure gradient entailing increased thoracic blood volume and reduced splanchnic blood volume, increasing right atrial and pulmonary artery pressure, as well as respective changes of pulmonary vascular resistance. Systemic vascular resistance decreases and renal function improves by increased central blood volume, decreased renal vasoconstriction, decreased activation of the sympathetic nervous system and renin-angiotensin–aldosterone system [18, 19]. In this study, a reliable prediction which patients will experience an increase in PSG was not possible. We therefore suggest short-term follow-up within the first week after TIPS after equilibration of portal hemodynamics.

The hepatic venous pressure gradient (HVPG) as used by the American Association for the Study of the Liver [20] is defined as difference between the pressure of the wedged hepatic vein and the free hepatic vein and is an indirect calculation of pressure gradients rather than a direct measurement [21]. Those HVPG levels have been discussed in clinical studies in order to find adequate cut-off levels (≤ 10, ≤ 12, or ≤ 20 mmHg) with respect to clinical outcome [20, 22–26]. However, these indirect pressure levels might not be compared with directly registered PSG values because of the different technical methods. This is particularly true for a TIPS cohort in whom vascular anatomy has been changed by creation of shunt flow. Furthermore, there have been concerns on whether to obtain the reference measurement from the right atrium, the hepatic veins, from a certain positions within an particular hepatic vein, or, alternatively, from the inferior vena cava as results may be different [27, 28]. In order to cover all these individual confounders, mean right atrial pressure was used as reference pressure in this study. Positioning the tip of the sheath in the right atrium and the catheter within the main portal vein is easy to perform and allows for standardized simultaneous pressure measurements before and after TIPS. In addition, factors like deep sedation, patient movement, and coughing or breathing during measurement may impact pressure levels [29–31]. These factors were overcome by standardized conditions as described above.

PSG, as defined here, is impacted by the whole vascular resistance between the portal vein and the right atrium and includes potential factors such as pre-sinusoidal pathologies, intrahepatic veno-venous shunts, or stenoses of the hepatic and caval vein [21, 23]. Finally, the PSG is affected by the compliance of the right atrium, increased atrial pressures, particularly in heart and pulmonary diseases which cause backpressure for venous return [15–17]. In this study, our suggested PSG cut-off of ≤ 8 mmHg at follow-up is challenging; therefore, the data had also been analyzed for a PSG cut-off of ≤ 10 and ≤ 12 mmHg. However, looking at reports on TIPS using PTFE stentgrafts, the immediate post-TIPS pressure gradients are often between PSG 7 and 9 mmHg, independent of the formally defined cut-off level of < 12 mmHg in the respective methods Sects. [32–34]. Insofar, the cut-off defined in our study (PSG ≤ 8 mmHg) is more an adaption to clinical practice.

Our study may be criticized as pathophysiological pressure data follow-up was not correlated with clinical data. Since clinical complications such as rebleeding and alleviation of ascites have been reported to correlate with PSG, we herein focused on this issue to point out the changes of porto-venous features and not the clinical sequelae. Another point of criticism may be that secondary alterations of the TIPS tract impacting shunt flow or remodeling of the TIPS tract by curving, kinking, and radial forces of the stent which may take longer than 4 days [35, 36]. Finally and most important, clinical outcome is influenced by the entity and progression of the underlying diseases, which was also beyond the scope of this study. Further studies should therefore investigate the portal hemodynamics and correlate with clinical outcome and re-intervention rates during mid-term and long-term follow-up.

In conclusion, pressure characteristics significantly changes within a few days after TIPS placement. Data obtained immediately after the TIPS procedure have only moderate predictive power for the future portal hemodynamics. Besides the absolute pressure reduction in the portal vein, the highly variable right atrial pressure changes were the main contributor to different pressure gradients. This includes the possibility that different portal vein pressures may result in the same pressure gradients and vice versa. Since pressure gradients have been reported to impact further clinical proceeding, studies are required to correlate detailed hemodynamic changes with clinical outcome.

## Supplementary Information

Below is the link to the electronic supplementary material.

Supplementary file1 (DOCX 49 KB)
