# Supplementary material for: Transjugular Portosystemic Stent Shunt: Impact of Right Atrial Pressure on Portal Venous Hemodynamics Within the First Week
Source: Cardiovasc Intervent Radiol. 2021 Dec 1;45(1):102–11. doi: 10.1007/s00270-021-03003-z (PMC8716358; doi:10.1007/s00270-021-03003-z)
Supplement: Supplementary file 1 — Supplementary file1 (DOCX 49 KB) [file 270_2021_3003_MOESM1_ESM.docx]

**Supplemental data and tables**

**Analysis of follow-up intervals and pressure changes:**

The median interval for short-term follow-up was 4 days. The majority of patients (110 of 124; 88.7%) received the follow-up within the first 7 days after TIPS. In 14 patients, TIPS control was performed beyond the first week after recovery from the intensive care unit (Suppl.Tab.1). Patients with TIPS control beyond the 4th or 7th day presented slightly increased PAP and PSG at follow-up. However, there was no statistical significance of the respective pressure changes during short-term follow-up (ΔPAPcontol-post, ΔRAPcontrol-post, and ΔPSGcontrol-post) between the shorter or longer follow-up period of more than 4 or 7 days compared to the earlier follow-up, respectively (Suppl.Tab.2).

| **Short-term follow-up (d)** | **Number of patients (n)** | **%** |
| --- | --- | --- |
| 2 | 24 | 19.4 |
| 3 | 36 | 29.0 |
| 4 | 24 | 19.4 |
| 5 | 14 | 11.3 |
| 6 | 9 | 7.3 |
| 7 | 3 | 2.4 |
| 8 | 3 | 2.4 |
| 9 | 1 | 0.8 |
| 10 | 2 | 1.6 |
| 11 | 1 | 0.8 |
| 13 | 1 | 0.8 |
| 14 | 2 | 1.6 |
| 19 | 1 | 0.8 |
| 25 | 1 | 0.8 |
| 32 | 1 | 0.8 |
| 34 | 1 | 0.8 |
| Total | 124 | 100.0 |

Suppl.Tab 1: Distribution of time-intervals at short-term follow-up. A total of 84 of 124 patients (67.7%) received TIPS control between day 2 and 4 after TIPS. A total of 110 of 124 patients (88.7%) underwent TIPS control within one week after TIPS. The remaining patients had a postponed follow-up because of a prolonged stay on the intensive care unit.

|  | TIPScontrol  ≤4d (n=84) | >4d  (n=40) | p | TIPScontrol  ≤7d (n=110) | >7d  (n=14) | p |
| --- | --- | --- | --- | --- | --- | --- |
| PVPcontrol | 14.0±4.8  (3 to 26) | 17.1±5.0  (8 to 29) | 0.001 | 14.4±4.7  (3 to 26) | 19.1±6.3  (10 to 29) | 0.001 |
| RAPcontrol | 5.9±4.4  (-2 to 19) | 7.6±4.6  (0 to 16) | 0.054 | 6.2±4.5  (-2 to 19) | 8.1±4.6  (1 to 16) | 0.137 |
| PSGcontrol | 8.1±3.1  (1 to 18) | 9.5±4.0  (3 to 20) | 0.038 | 8.2±3.2  (1 to 18) | 11.0±4.7  (4 to 20) | 0.005 |
| ΔPVP control-post | -3.0±4.7  (-14 to 7) | -1.7±4.1  (-11 to 9) | 0.115 | -2.7±4.5  (-14 to 7) | -2.0±4.5  (-8 to 9) | 0.598 |
| ΔRAP control-post | -5.4±5.2  (-21 to 4) | -5,1±4.45  (-19 to 6) | 0.740 | -5.1±5.0  (-21 to 6) | -6.3±4.9  (-19 to 2) | 0.418 |
| ΔPSG control-post | 2.3±3.2  (-8 to 12) | 3.4±3.5  (-5 to 12) | 0.103 | 2.5±3.3  (-8 to 12) | 4.3±3.5  (-1 to 11) | 0.053 |

Suppl.Tab.2: Portal pressure values and portal pressure changes at short-term follow-up after TIPS placement. TIPS control within 4 days versus >4days follow-up. TIPS control within ≤ 7 days versus >7 days follow-up, p significance, T-Test.

**Subgroup analysis:**

In a subgroup analysis of those 105 patients who primarily met the cut-off immediately after TIPS placement (n=105), only 60 patients still met this cut-off at follow-up and whereas 45 patients failed (Tab3a). Patients who failed the cut-off presented an increased PVP before and after TIPS, as well as an increased PSG immediately after TIPS. However, there were no statistically significant differences of the individual pressure changes between both subgroups. With respect to clinical data, patients of group II showed slightly reduced Albumin levels, but no statistical differences with respect to demographics, clinical stage, indication for TIPS, or other laboratory tests (Suppl.Tab.3).

| **Demographics** | **Group I (PSGcontrol≤8mmHg)** | **Group II**  **(PSGcontrol>8mmHg)** | **p** |
| --- | --- | --- | --- |
| **Patients (n)** | **60** | **45** |  |
| Age mean ±SD (years) | 60.9±12.8 | 56.7±13.1 | 0.1 * |
| Male / Female (n) | 40/20 | 25/20 | 0.246 ** |
| **Clinical stage** |  |  |  |
| ChildPugh (A/B/C) | 10/34/16 | 1/31/13 | 0.055** |
| Child-Pugh points (Median/Q1/Q3) | 8/6.5/9.5 | 9/8/10 | 0.144 *** |
| MELD (Median/Q1/Q3) | 12.5/9.5/15.5 | 12/9.2/14.8 | 0.830*** |
| NaMELD (Median/Q1/Q3) | 15.5/11.3/19.8 | 17/13/22 | 0.091*** |
| **Clinical findings** |  |  |  |
| History of HE (no/grade 1-2/grade 3-4) | 47/10/3 | 37/4/4 | 0.407* |
| History of HRS (no/yes) | 39/21 | 43/11 | 0.172* |
| History of SBP (no/yes) | 52/8 | 35/10 | 0.175* |
| **Laboratory test** |  |  |  |
| INR | 1.3/1.2/1.4 | 1.3/1.2/1.4 | 0.388*** |
| Creatinine (mg/dl) | 1.07/0.77/1.37 | 0.97/0.56/1.4 | 0.175*** |
| Bilirubin (mg/dl) | 1.2/0.54/1.86 | 1.3/0.53/2.07 | 0.290*** |
| Albumin (g/l) | 28/24/32 | 26/23.2/28.8 | 0.01*** |
| Thrombocytes (n/µl) | 110/58/162 | 128/69/188 | 0.184*** |
| **Clinical indication for TIPS** |  |  |  |
| Refractory ascites / hydrothorax | 36 | 32 |  |
| Refractory ascites +history of bleeding | 5 | 2 |  |
| Variceal bleeding | 17 | 11 |  |
| Variceal bleeding + ascites | 2 | 0 | 0.437* |
| **Portal hemodynamics** |  |  |  |
| PVPpre | 22.9±4.1 (13 to 31) | 25.2±4.8 (16 to 42) | 0.011** |
| RAPpre | 7.5±3.9 (-3 to 20) | 9.2±5.4 (0 to 27) | 0.07** |
| PSGpre | 15.4±4.8 (3 to 31) | 16.0±4.9 (6 to 32) | 0.521** |
| PVPpost | 16.0±4.4 (6 to 29) | 18.4±4.1 (12 to 31) | 0.005** |
| RAPpost | 11.6±4.3 (2 to 26) | 12.5±4.4 (6 to 24) | 0.295** |
| PSGpost | 4.4±1.8 (1 to 8) | 6.0±1.9 (0 to 8) | 0.000** |
| ΔPVPpost-pre | -6.9±3.9 (-18 to 2) | -6.8±3.4 (-16 to 2) | 0.843** |
| ΔRAPpost-pre | 4.1±3.5 (-2 to 21) | 3.3±3.9 (-8 to 11) | 0.297** |
| ΔPSGpost-pre | -11.0±4.6 (-28 to -1) | -10.1±4.7 (-25 to 1) | 0.324** |

Suppl.Tab.3: Demographics and clinical data of 105 patients who fulfilled the cut-off of PSG ≤8mmHg at immediately after TIPS completion. Group I: patients who fulfilled the target PSG≤8mmHg at short-term follow-up. Group II: patients who failed the target PSG at short-term follow-up. HE: Hepatic encephalopathy. HRS: Hepato-renal syndrome. SBP (Spontaneous bacterial peritonitis). P significance * ChiSquare Test, ** T-Test, *** Mann-Whitney-U-Test
